# Temperature Dependence of Tensile Properties and Deformation Behavior in Highly Strong Heat-Elongated Polypropylene

**DOI:** 10.3390/polym17243238

**Published:** 2025-12-05

**Authors:** Karin Onaka, Hiromu Saito

**Affiliations:** Department of Applied Chemistry, Tokyo University of Agriculture and Technology, Koganei-shi 184-8588, Japan; s237824q@st.go.tuat.ac.jp

**Keywords:** polypropylene, tensile strength, DMA, DSC, small-angle X-ray scattering, elongation, high temperature, deformation behavior

## Abstract

We investigated the tensile properties and deformation behavior at various temperatures of highly strong heat-elongated polypropylene (PP), in which stacks of crystalline lamellae are macroscopically arranged in the elongated direction and lamellae are connected by thin fibrils. The elastic modulus *E*′ and the αc-relaxation temperature for the onset of crystalline chain motion, obtained through dynamic mechanical analysis, were higher in the heat-elongated than the unelongated PP, indicating the suppression of crystalline chain motion. The heat-elongated PP deformed beyond the yield point at high temperatures above the αc-relaxation point, and it exhibited high tensile stress; e.g., the yield stress was 60 MPa at 120 °C, which was 7.5 times higher than that of the unelongated PP. Small-angle X-ray scattering intensity patterns changed from layered to diffuse, and DSC thermograms showed that melting peak position shifted to lower temperatures when stretching at small strains at various temperatures. The results suggest that lamella fragmentation occurs under small strains at various temperatures. Thus, the good high-temperature strength of the heat-elongated PP is due to the fragmentation of lamellae during small-strain stretching and the suppression of crystalline chain motion by thin crystalline fibrils connected to the lamellae.

## 1. Introduction

Crystalline polymers such as polypropylene (PP) are used extensively in various applications, including consumer products, automotive parts, medical devices and film packaging, owing to their light weight, high temperature and chemical resistance, ease of processing and low cost [1,2,3,4,5]. PP use extends to composite materials, including concrete/PP fiber composites [6,7], recycling-friendly self-reinforced PP composites [8], and PP-based synthetic paper [9]. Numerical studies have been conducted on the tensile properties and structural evolution of crystalline polymers during stretching [10,11,12,13,14]. Tensile stress increases steeply with strain until reaching a maximum value at the yield point due to interlamellar separation and slip in the initial stretching stage [15]. Then, due to lamella fragmentation and void formation, plastic deformation occurs, and the stress increases due to strain hardening as a result of fibrillation in the plastic region [12,16,17]. Fibrillation of lamellae is evaluated by changes in the melting peak of a differential scanning calorimetry (DSC) thermogram [18,19,20]; yield stress depends on crystallinity, lamellar thickness [21,22] and spherulite size [23]; and strain hardening depends on the density of chain entanglement in the amorphous region [11]. Tensile stress increases with increasing strain rate [24]. Void formation during stretching also contributes to stress–strain behavior and structure evolution [25,26,27,28,29,30]. Large-scale molecular dynamics simulations assuming various lamellar structures also used to derive stress–strain curves [31,32].

As the temperature increases, tensile stresses such as yield stress typically decrease due to enhanced chain motion [12,21,22,33,34,35,36,37,38]. By elevating the temperature, chain motion is enhanced. The αa-relaxation temperature signals the onset of amorphous chain motion, related to the glass transition temperature, and crystalline chain motion is enhanced at the αc-relaxation temperature, as evaluated via dynamic mechanical analysis (DMA) [34,39,40,41]. The deformation behavior changes at the αa- and αc-relaxation temperatures due to changes in chain motion that contribute to deformation of crystalline and amorphous regions [18,37]. Tensile strength and dynamic modulus change at around the αc-relaxation temperature due to changes in crystallite stability and amorphous chain strength [34].

It is well known that crystalline polymers’ structures and tensile properties can be manipulated by elongation [13,42,43,44,45,46]. For instance, the shish-kebab structure, consisting of extended crystalline chains (shish) assembled with folded-chain crystalline lamellae (kebab), is formed by orientation crystallization [47,48]. The tensile strength of shish-kebab crystals increases with increased crystallinity and interlocking of the crystals, which decrease deformability [45,46,49]. Macroscopically arranged stacked layer structures consisting of hard crystalline lamellae and soft amorphous layers, obtained by heat elongation, exhibit high strength due to yielding suppression, which prevents the lamella fracture and void formation [50,51]. Impact strength is improved by stretching and adding nucleation agent [52]. Tensile modulus and strength improve after biaxial heat elongation due to the formation of a nanosized fiber-like network and reduction in lamellar size [53]. Large-scale molecular dynamics simulations reproduce the improved tensile properties caused by decreased lamellar size [54]. Highly strong and transparent film was obtained due to the refinement of lamellae by heat elongation in crystallized cyclo olefin polymers [55]. It has also been reported that the increase in stress during strain hardening is enhanced by the cross-linked crystal network [44,56], as observed in elastomers due to their topological network structure [57]. Although there are various methods and concepts for strengthening by elongation, the process is usually only evaluated at room temperature. Therefore, the temperature dependences of highly strong heat-elongated crystalline polymers remain unclear, even though high-temperature tensile properties are significant for understanding strengthening mechanisms and applications.

In this paper, to understand the strengthening ability of uniaxially heat-elongated crystalline polymers at high temperatures, we investigated the temperature dependence of their tensile properties using tensile testing and DMA. We chose polypropylene (PP) as the crystalline polymer for this study, because its αa- and αc-relaxation temperatures can be detected, allowing us to analyze crystalline and amorphous chain motion at various temperatures [34,39]. Small-angle X-ray scattering (SAXS) and DSC measurements were discussed to clarify the characteristic deformation behavior of the heat-elongated PPs at different temperatures by comparing the values with those of the unelongated PP.

## 2. Materials and Methods

### 2.1. Materials

The polypropylene (PP) used in this study was isotactic polypropylene supplied by Prime Polymer Co., Ltd., Tokyo, Japan (Prime Polypro J-703GR), with a melt flow rate (MFR) of 9.6 g/10 min.

PP pellets were compression-molded at 200 °C for 3 min using a hot-press machine (11FD, Imoto Machinery Co., Ltd., Kyoto, Japan) and then quenched in a cold water bath to obtain PP film with a thickness of about 230 μm. The quenched PP film was annealed at 120 °C for 1 h in an oven (DVS403, Yamato Scientific Co., Ltd., Tokyo, Japan) and then cooled to obtain unelongated annealed PP film.

Heat-elongated PP film was obtained by heating the quenched film to 120 °C and uniaxially elongating it at this temperature up to an elongation ratio of 600% at a constant rate of 50 mm/min using a heat stretching apparatus (Figure S1: Taiatsu Techno Corporation, Tokyo, Japan). The heat-elongated specimens were immediately cooled to room temperature while under tension to prevent large orientation relaxation.

### 2.2. Methods

#### 2.2.1. SAXS Measurement

Small-angle X-ray scattering (SAXS) experiments were performed using the NANO-Viewer system (Rigaku Co., Ltd., Tokyo, Japan). Cu-Ka radiation with a wavelength of 0.154 nm was generated at 46 kV and 60 mA, collimated by a confocal max-flux mirror system. SAXS intensity images were measured at room temperature with an exposure time of 1 h using an imaging plate (IP) (BAS-SR 127, Fujifilm Corp., Tokyo, Japan) as a two-dimensional detector. The X-ray was radiated at the midpoint of the stretched area, and the obtained scattering images were transformed into text data by an IP reading device (RAXIA-Di, Rigaku Co., Ltd., Tokyo, Japan). The scattering intensities were corrected for specimen thickness, beam transmittance and background scattering.

#### 2.2.2. Tensile Tests at Different Temperatures

Tensile tests were performed at different temperatures using a heat stretching apparatus (Figure S1; Taiatsu Techno Corporation, Tokyo, Japan) equipped with a tensile load cell (LUX-B-50N-ID-P, Kyowa Electronic Instrument Co., Ltd., Tokyo, Japan) and a sensor interface (PCD-4308, Kyowa Electronic Instrument Co., Ltd.) at a stretching speed of 10 mm/min. Dumbbell-shaped film specimens were prepared using a die-cutter, in accordance with ASTMD 1708. The length and width of the specimens were 35.0 mm and 5.0 mm, respectively. The stretching temperature (*T*s) ranged from room temperature to 120 °C, and the specimens were thermally stabilized for 5 min before stretching. After the stretching, the specimens were immediately cooled to room temperature while under tension to prevent large orientation relaxation. The stretching direction was parallel to the elongation direction of the films. Three tensile tests were performed for each data set, and the data set with the largest break strain is presented, because these specimens are least susceptible to damage and defects.

#### 2.2.3. DMA Measurement

Dynamic mechanical analysis (DMA) was carried out using a DMA1 (Mettler Toledo, Columbus, OH, USA) in tension mode. The tested specimens were 5 mm wide with a gap distance of 5 mm, and they were swept from −50 to 140 °C at a heating rate of 2 °C/min and a constant oscillatory frequency of 1 Hz. The storage modulus *E*′ and loss modulus *E*″ were obtained as functions of temperature.

#### 2.2.4. DSC Measurement

Dynamic scanning calorimetry (DSC) measurements were performed on specimens at a heating rate of 10 °C/min in a nitrogen atmosphere using a DSC-Q200 (TA Instruments, New Castle, DE, USA). The weight of the test specimens was about 3 mg.

## 3. Results and Discussion

### 3.1. Strengthening by Heat Elongation

**Figure 1:**
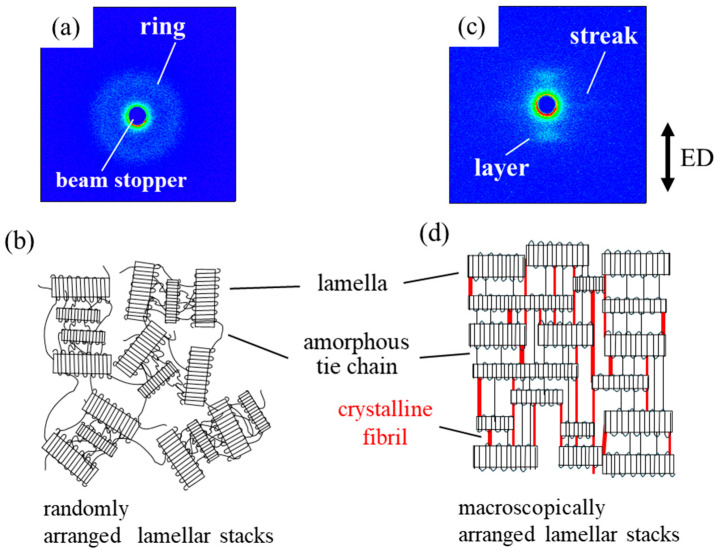
SAXS images (**a**,**c**) and their corresponding structure models (**b**,**d**): (**a**,**b**) unelongated PP, (**c**,**d**) heat-elongated PP. ED is elongated direction.

Figure 1 shows the small-angle X-ray scattering (SAXS) images of the unelongated and heat-elongated polypropylene (PP). Here, the unelongated sample was obtained by annealing quenched PP at 120 °C for 1 h, and the heat-elongated PP was obtained via uniaxial heat elongation of the quenched material at 120 °C up to an elongation ratio of 600%. The unelongated PP exhibited an isotropic ring pattern (Figure 1a), indicating a randomly arranged stacked structure consisting of crystalline lamellae and amorphous layers (Figure 1b). On the other hand, the heat-elongated PP exhibited a layer pattern on the meridian that was perpendicular to the direction of elongation (Figure 1c). This indicates a macroscopically arranged stacked structure consisting of short crystalline lamellae and amorphous layers, in which stacked lamellae are arranged in the elongated direction, with the longitudinal direction of the lamellae perpendicular to the elongation (Figure 1d). A weak streak pattern was observed on the equator, suggesting the presence of a thin fibrillar structure extending in the elongated direction.

Table 1 summarizes the structure parameters of the unelongated and heat-elongated PP. As determined by DSC, the melting enthalpy (Δ*H*), related to crystallinity, and the melting temperature (*T*m) of the heat-elongated PP were 1.4 times and 6 °C higher, respectively, than those of the unelongated sample. The periodic distance between adjacent lamellae and the lamellar and amorphous layer thicknesses, as obtained by the correlation function of the SAXS intensity profiles, indicated that the thicknesses of both the lamellae and amorphous layers increased via heat elongation; i.e., the thicknesses of the unelongated and heat-elongated lamellae were 9.1 nm and 10.3 nm, respectively, and the amorphous layer thicknesses were 5.2 nm and 7.1 nm, respectively.

**Figure 2:**
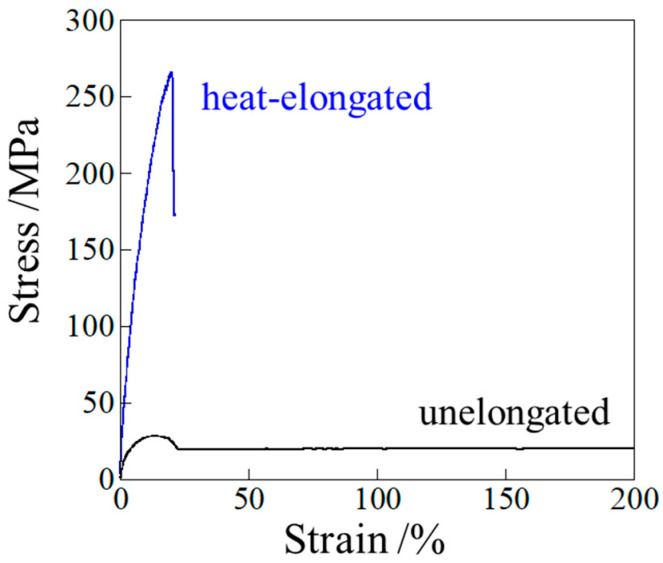
Stress–strain curves of the unelongated and heat-elongated PP, measured at room temperature.

Figure 2 shows the stress–strain curves of the unelongated and heat-elongated PP measured at room temperature. Here, the tensile test for the heat-elongated PP was performed with the stretching direction parallel to the elongation and perpendicular to the longer direction of the lamellae. In the unelongated PP, the stress increased linearly with strain at small strains, reaching a maximum at the yield point after a slight decrease in stress. Yielding is considered to occur due to the fragmentation of lamellar stacks without lamellar fragmentation [58]. After yielding, the stress in the unelongated sample increased gradually with strain due to strain hardening until breaking. On the other hand, in the heat-elongated PP, the stress increased steeply with strain up to 20%, with the sample breaking at around the yield point. The yield stresses of the unelongated and heat-elongated PP were 28 MPa and 266 MPa, respectively; i.e., the yield stress of the heat-elongated PP was approximately 9.5 times greater than that of the unelongated sample. Thus, PP heat elongation caused drastic strengthening. As indicated by the SAXS results shown in Figure 1, the highly strong heat-elongated PP has a macroscopically arranged stacked structure consisting of hard crystalline lamellae and soft amorphous layers, similar to those observed in heat-elongated high-density polyethylene (HDPE) and thermoplastic polyurethane (TPU) [50,51]. The stress at break of the PP was higher than those measured for HDPE and TPU, i.e., 266 MPa for PP, 170 MPa for HDPE and 136 MPa for TPU.

### 3.2. Dynamic Mechanical Behavior

**Figure 3:**
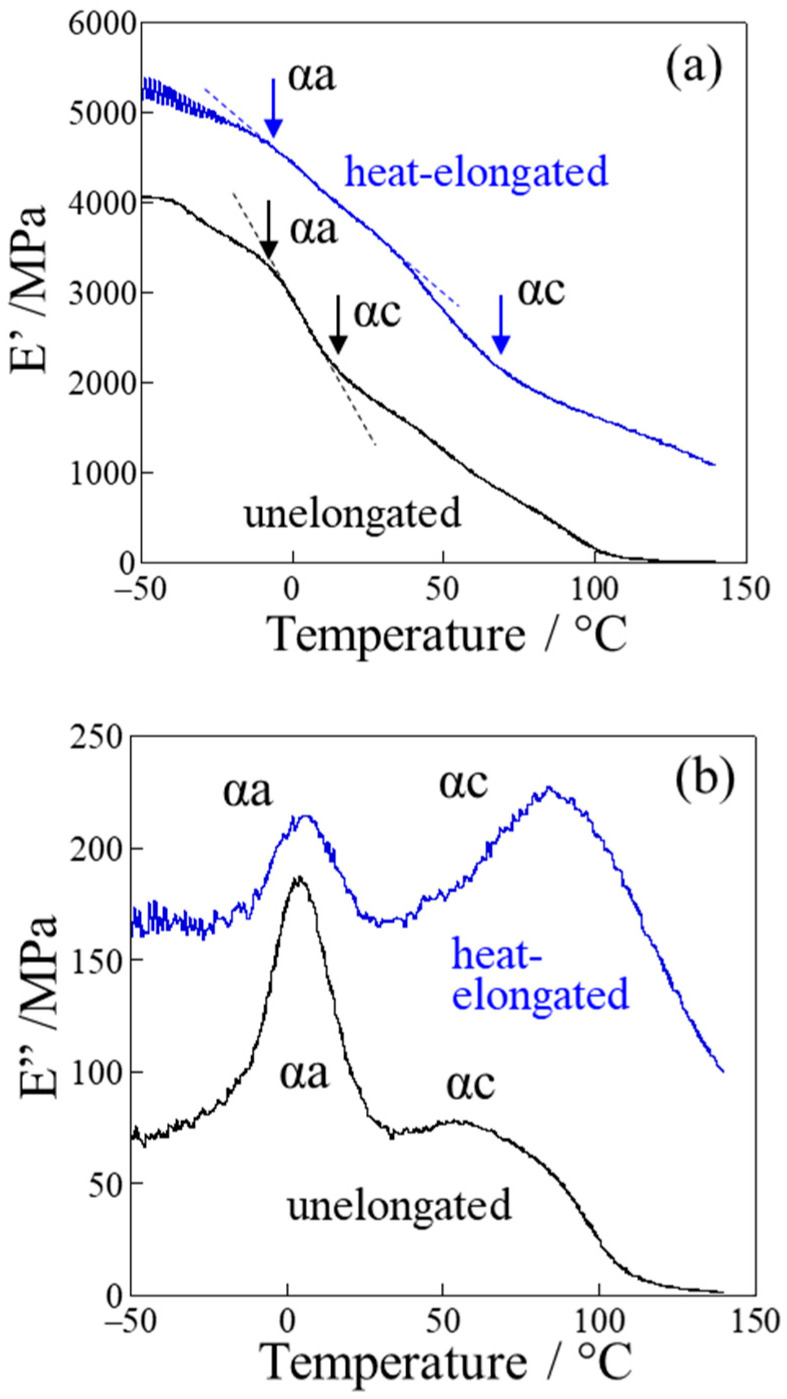
DMA thermograms of the unelongated and heat-elongated PP: (**a**) storage modulus *E*′; (**b**) loss modulus *E*″.

Figure 3 shows the variations in elastic modulus (*E*′) and loss modulus (*E*″) with temperature, obtained by dynamic mechanical analysis (DMA) at a frequency of 1 Hz. Two inflection points and two peaks were observed in *E*′ and *E*″, respectively, in both the unelongated and heat-elongated PP. These are related to two types of relaxation: αa-relaxation at lower temperature and αc-relaxation at higher temperatures. αa-relaxation is characteristic of large-scale amorphous chain motion [59,60] that freezes at the glass transition temperature *T*_g_ [61]. On the other hand, αc-relaxation is characteristic of crystalline chain motion, caused by crystalline chain slippage [60,62] and the diffusion of conformational defects within the crystals [40,59,63].

The *E*′ of the heat-elongated PP was much larger than that of the unelongated material, exhibiting a larger value across the entire temperature range, i.e., from −50 °C to 140 °C (Figure 3a). In the unelongated PP, the *E*′ decreased gradually with temperature before dropping steeply from the αa-relaxation temperature of around −10 °C to the αc-relaxation temperature around 15 °C. Here, the αa-relaxation temperature represents the onset of amorphous chain motion, while the αc-relaxation temperature represents the onset of crystalline chain motion [34,39,40,41]. The αc-relaxation temperature of the heat-elongated PP was observed at around 70 °C, 55 °C higher than that of the unelongated PP. This increase in αc-relaxation temperature in the heat-elongated PP is attributed to the suppression of crystalline chain motion.

The αa-relaxation peak observed in the *E*″ of the heat-elongated PP was smaller than that of the unelongated sample (Figure 3b), due to the higher crystallinity of the heat-elongated PP, shown in Table 1, and the suppression of amorphous chain motion. The αa-relaxation peak of the heat-elongated PP had no tail on the lower-temperature side, while a tail was observed in the unelongated PP. This result also suggests the suppression of amorphous molecular motion in the heat-elongated material at around the αa-relaxation temperature, causing a smaller decrease in *E*′ in the heat-elongated PP at around the αa-relaxation temperature—from −40 °C to 15 °C (Figure 3a). The peak area of the αc-relaxation peak was much smaller in the heat-elongated than the unelongated PP, and the peak temperature of the heat-elongated PP was the higher of the two (Figure 3b). The shift in αc-relaxation peak to a higher temperature is considered to be due to the larger crystal connectivity and crystalline length, requiring more thermal energy [34,64].

At temperatures higher than the αc-relaxation temperature, the decrease in *E*′ was suppressed in the heat-elongated PP up to 140 °C, while it decreased to nearly zero at around 100 °C in the unelongated PP (Figure 3a). The suppression of the decrease in *E*′ at high temperatures in the heat-elongated PP can be attributed to the large crystal connectivity as a result of its crystalline lamellae connected by thin crystalline fibrils, as indicated by the SAXS results in Figure 1c,d. In contrast, the unelongated PP shows lower crystal connectivity due to the crystalline lamellae only being connected by amorphous tie chains acting as physical cross-links.

### 3.3. Stress–Strain Behavior at Different Temperatures

**Figure 4:**
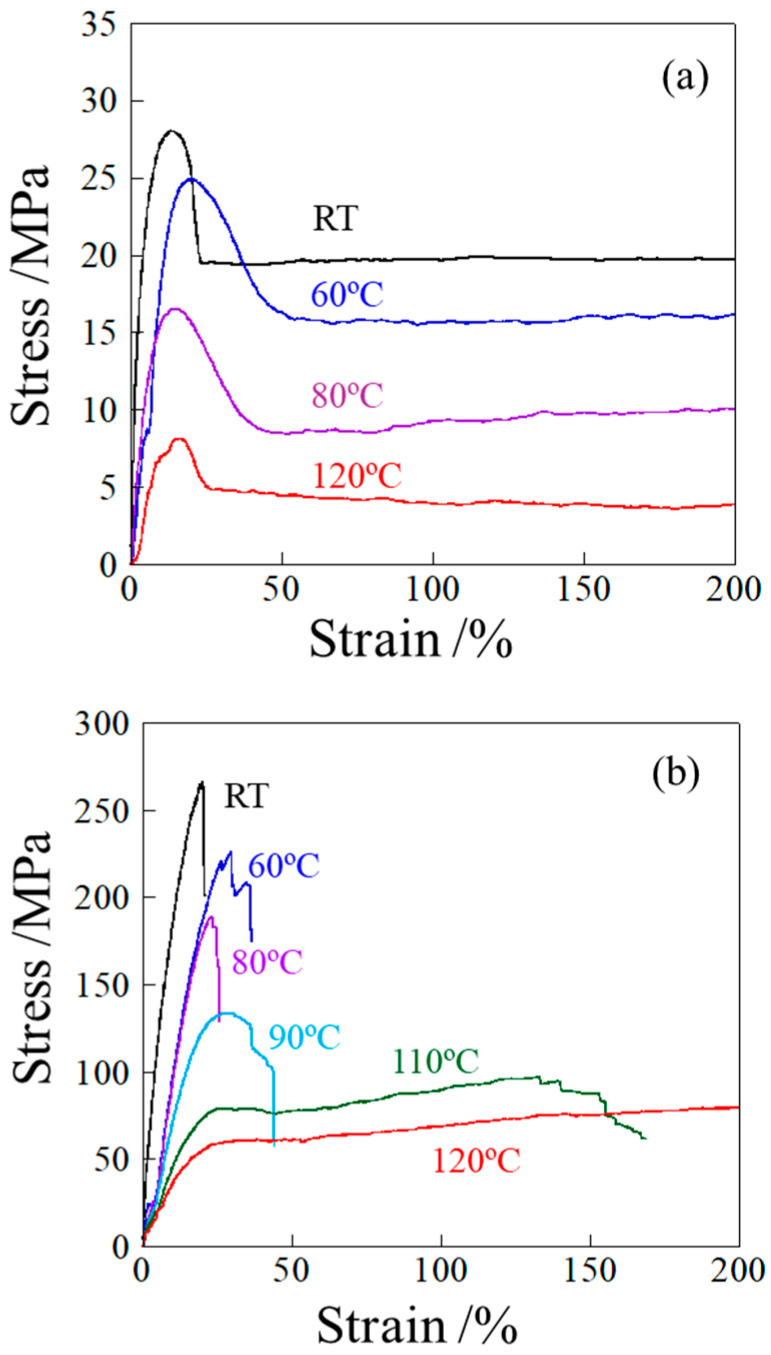
Stress–strain curves measured at different temperatures: (**a**) unelongated PP; (**b**) heat-elongated PP.

Figure 4 shows the stress–strain curves of the unelongated and heat-elongated PP measured at various temperatures (*T*s). The stress decreased continuously with increasing the *T*s in both materials. There was no significant difference in stress–strain curve shape for the unelongated PP at different *T*s; i.e., stress increased almost linearly with strain in the small-strain region, reaching a maximum at the yield point and then gradually increasing with strain after yielding due to strain hardening until breaking (Figure 4a). On the other hand, the stress–strain behavior of the heat-elongated PP changed significantly at around the αc-relaxation temperature (Figure 4b). The heat-elongated PP broke at around the yield point below the αc-relaxation temperature, while it deformed beyond the yield point above the αc-relaxation temperature. The decrease in stress after the yield point was slight at 110 °C and 120 °C in the heat-elongated PP (Figure 4b), while it was significant in the unelongated PP (Figure 4a).

Even at temperatures above the αc-relaxation temperature *T*_αc_, the heat-elongated PP exhibited a high yield stress (Figure 4b, greater than 130 MPa at 90 °C (*T* − *T*_αc_ = 20 °C) and above 60 MPa at 120 °C (*T* − *T*_αc_ = 50 °C), while yield stress was below 30 MPa at around room temperature (*T* − *T*_αc_ = 10 °C) and lower than 25 MPa at 60 °C (*T* − *T*_αc_ = 45 °C) in the unelongated PP. The results indicate that the yield stress was much higher in the heat-elongated than the unelongated PP in the *T* − *T*_αc_ range of around 20 °C to 50 °C. Thus, the high strength of the heat-elongated PP cannot be explained only by the suppression of crystalline motion due to the shift in *T*_αc_ towards higher temperatures, but also by the characteristic deformation behavior, as demonstrated in Section 3.4.

**Figure 5:**
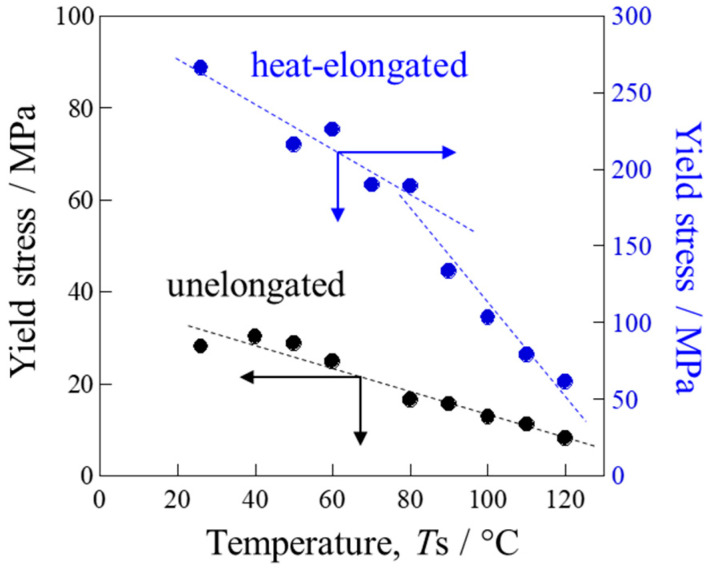
Yield stress of the unelongated and heat-elongated PP as a function of measuring temperature, *T*s.

The yield stresses presented in Figure 4 are shown in Figure 5 as a function of measuring temperature, *T*s. In the heat-elongated PP, when tested below the αc-relaxation temperature, the yield stress obtained was lower than the true yield stress because the specimen might break before reaching its maximum stress at the yield point. The yield stress decreased almost linearly with *T*s in the unelongated PP, for which the αc-relaxation temperature was around 15 °C. On the other hand, bending was observed in the heat-elongated PP at around 80 °C, close to the αc-relaxation temperature of 70 °C as evaluated from *E*′ in Figure 3a. The slope was gentle below the αc-relaxation temperature due to the presence of only amorphous chain motion, while it was steep above the αc-relaxation temperature due to simultaneous amorphous and crystalline chain motion. Due to the additional contribution of crystalline chain motion above the αc-relaxation temperature, the heat-elongated PP deformed beyond the yield point, as shown in Figure 4b. The yield stress of the heat-elongated PP at 120 °C was much larger than that of the unelongated PP at 25 °C, at 60 MPa and 28 MPa, respectively. On the contrary, as shown in Figure 3a, the elastic modulus *E*′ of the heat-elongated PP at 120 °C was much lower than that of the unelongated PP at 25 °C, at 1400 MPa and 1800 MPa, respectively. This opposite result is attributed to the characteristic deformation behavior of the heat-elongated PP, as demonstrated in the next section.

### 3.4. Deformation Behavior

**Figure 6:**
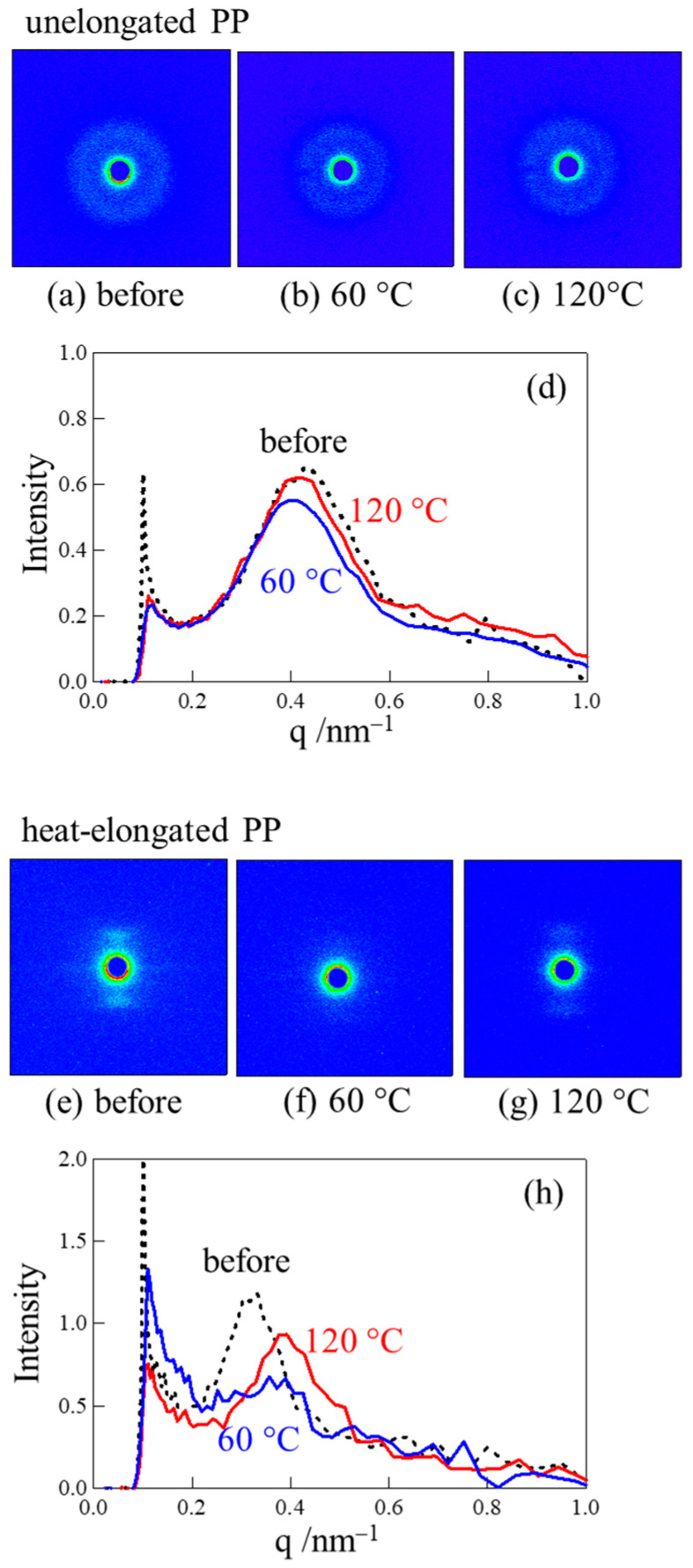
SAXS images and intensity profiles of (**a**–**d**) unelongated and (**e**–**h**) heat-elongated PP obtained after stretching at 10% strain under temperatures of 60 °C and 120 °C.

Figure 6 shows the small-angle X-ray scattering (SAXS) images and the intensity profiles of the unelongated and heat-elongated PP obtained after stretching at a small strain of 10% below the yield point at different temperatures. In the unelongated PP, the isotropic ring pattern shown in Figure 1a exhibited no significant change when stretched at 60 °C or 120 °C (Figure 6a–c). As can be seen from the SAXS patterns, no significant intensity profile changes occurred by stretching at a small strain of 10% at both 60 °C and 120 °C, with a sharp peak remaining despite the stretching (Figure 6d). These results indicate that no significant structural change occurred under small strain below the yield point due to elastic deformation caused by interlamellar slip and fragmentation of lamellar stacks without lamellar fragmentation [12,16,17].

On the other hand, in the heat-elongated PP, the layer pattern of the macroscopically arranged lamellar stacks disappeared when stretched at a small strain of 10% at 60 °C, which was below the αc-relaxation temperature (Figure 6e,f). Alongside the change in scattering pattern from layered to diffuse, the sharp intensity peak changed to a broad unclear peak (Figure 6h). These results suggest that plastic deformation occurs due to lamellar fragmentation, though it is generally considered that this process does not occur below the yield point, as demonstrated by the unelongated PP in Figure 6a–d. A structural change caused by plastic deformation under small strain was also observed at a higher temperature of 120 °C, above the αc-relaxation temperature. Here, the layer pattern changed to a diffuse layer one (Figure 6e,g), and the sharp intensity peak became broader and shifted to a higher scattering vector, *q* (Figure 6h). The periodic distance between neighboring lamellae, *d*, derived from *d* = 2π/*q*, changed from 17.4 nm to 14.8 nm due to stretching, suggesting lamellar refinement by means of fragmentation. Thus, the high strength of the heat-elongated PP—a high yield stress of 225 MPa at 60 °C and 60 MPa at 120 °C—could result from plastic deformation caused by lamellar fragmentation. This deformation mechanism differs from that of highly strong heat-elongated high-density polyethylene and polyurethane, in which lamellae are not fragmented by stretching under small strain [50,51]. Such fragmentation is also not observed in PP spherulites [23].

**Figure 7:**
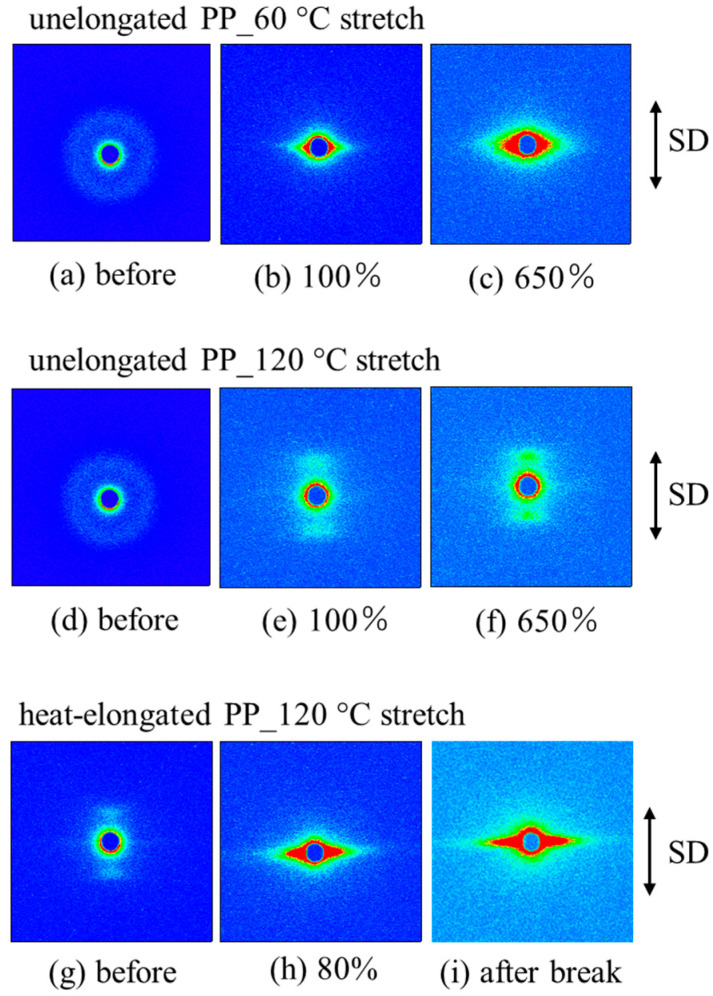
SAXS images of (**a**–**f**) unelongated and (**g**–**i**) heat-elongated PP obtained after stretching at different strains beyond the yield point and at different stretching temperatures of 60 °C and 120 °C.

Figure 7 shows the SAXS images of the unelongated and heat-elongated PP after stretching at different temperatures and large strains beyond yield points. In the unelongated PP, the isotropic ring pattern observed before stretching changed to a streak pattern due to stretching at 60 °C after yielding above 100% strain, indicating a structural change in the plastic region from a randomly arranged stacked lamellar structure to a fibrillar one (Figure 7a–c), induced by lamellar rearrangement and fragmentation [11,15,16,17,65,66]. On the other hand, stretching at 120 °C changed the isotropic ring pattern to a layer pattern on the meridian and a weak streak pattern on the equator, indicating a change from a randomly arranged stacked lamellar structure to a macroscopically arranged stacked lamellar one connected by thin crystalline fibrils, as shown in Figure 1c,d (Figure 7d–f). In the heat-elongated PP, stretching at 120 °C, which was above the αc-relaxation temperature, changed the layer pattern to a strong streak pattern, indicating that the macroscopically arranged stacked lamellar structure changed to a fibrillar one in the plastic region after yielding (Figure 7g–i). On the other hand, the heat-elongated PP was fractured before yielding by stretching at 60 °C, which was below the αc-relaxation temperature.

**Figure 8:**
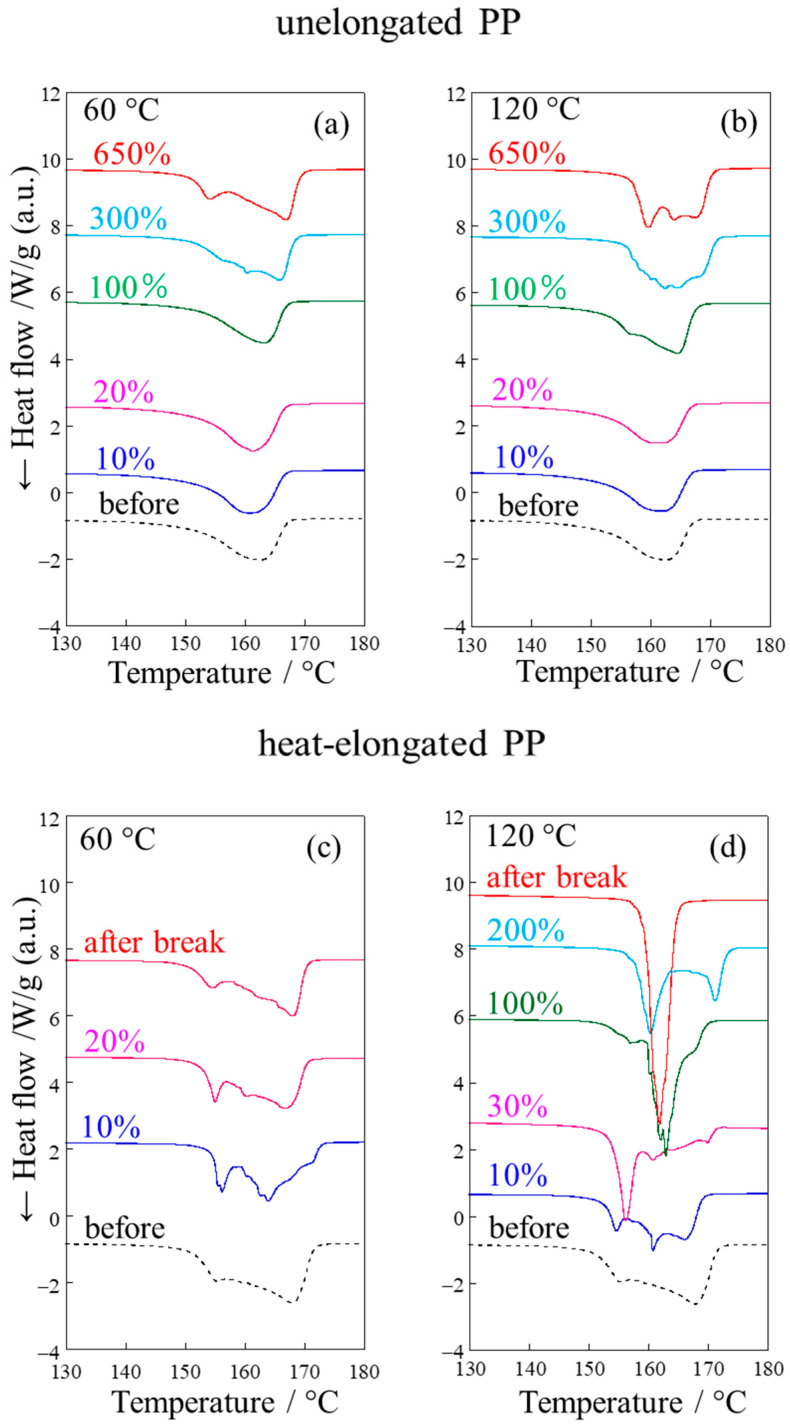
DSC thermograms for melting peak of (**a**,**b**) unelongated and (**c**,**d**) heat-elongated PP obtained after stretching at various strains and temperatures of (**a**,**c**) 60 °C and (**b**,**d**) 120 °C.

Figure 8 shows the DSC thermograms of the melting peaks of the unelongated and heat-elongated PP obtained after stretching at various strains and temperatures. In the unelongated PP, a single peak was observed before stretching, and no significant change was observed after stretching at small strains below 20% at either 60 °C or 120 °C (Figure 8a,b). These results indicate that no significant structural change in lamellae occurred before yielding due to elastic deformation without lamellar fragmentation, as suggested by the SAXS results shown in Figure 6a–d. However, significant changes occurred at large strains above 100% and 300% at 60 °C and 120 °C, respectively, with the single peak changing to two or more peaks at large strains due to fibrillation that occurs during plastic deformation, as described in detail in reference [18].

In the heat-elongated PP, two peaks were observed before stretching due to the co-existence of thicker lamellae and thinner crystalline fibrils (Figure 8c,d). The shape of the two peaks changed under stretching at small strains below 20% at both 60 °C and 120 °C, suggesting a change in lamellar structure due to plastic deformation despite the stretching being below the yield point. The melting peak changed at a much smaller strain in the heat-elongated than the unelongated PP, indicating that the change in lamellar structure is accelerated in the heat-elongated PP. The peak positions shifted to lower temperatures by stretching, suggesting a decrease in lamellar size. These results confirm lamellar refinement by fragmentation due to stretching at small strains below the yield point, as suggested by the SAXS results shown in Figure 6e–h. The multiple peaks changed to two sharp peaks and then to a sharp single peak beyond the yield point due to fibrillation during plastic deformation at large strains above 100% at 120 °C, above the αc-relaxation temperature. Thus, two types of crystallites with different thicknesses existed in the heat-elongated PP—thicker lamellae and thinner crystalline fibrils. The change in lamellar structure was accelerated, and lamellae were fragmented during stretching at small strains below the yield point. Such characteristic small-strain plastic deformation could result in the high yield stress of the heat-elongated material, 9.5 times higher than that of the unelongated PP.

### 3.5. Deformation Mechanism

**Figure 9:**
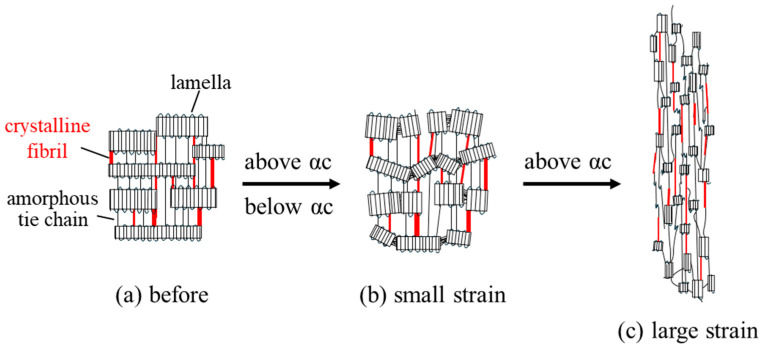
Schematic illustration of the structural change in the heat-elongated PP during stretching: (**a**) before stretching; (**b**) stretching at small strain below yield point; (**c**) stretching at large strain above yield point.

Figure 9 shows a schematic illustration of the deformation mechanism of highly strong heat-elongated PP during stretching, as suggested by the above results. The heat-elongated material exhibits a lamellar stack consisting of crystalline lamellae and amorphous layers that are macroscopically arranged in the elongation direction, with the stacked lamellae connected by thin crystalline fibrils and taut amorphous tie chains (Figure 9a). During small-strain stretching below the yield point, the lamellae in the stacks are fragmentated and refined (Figure 9b), with lamellar fragmentation possibly caused by crystalline chain within the lamellae. The fragmentation of lamellae at small strains causes high yield stress, as shown in Figure 4b, requiring large amounts of stress for deformation because of the suppression of crystalline chain motion and slippage in the heat-elongated PP, as indicated by the DMA results in Figure 3. Large amounts of stress cause crystalline chains to slip at small strains, resulting in the small-strain fragmentation of lamellae. Despite this lamellar fragmentation, fracture of the specimen via the breaking of the stacked lamellar structure is suppressed due to the presence of thin crystalline fibrils and taut amorphous tie chains that connect to fractured lamellae, which increases the break strain up to about 20%, resulting in high strength.

Below the αc-relaxation temperature, breaking a stacked lamellar structure by high applied stress induces a fracture at around the yield point before the specimen deforms into a fibrillar structure due to the suppressed crystalline chain molecular motion in the stiff lamellae obtained by heat elongation. On the other hand, above the αc-relaxation temperature, the stacked lamellar structure simply deforms into a fibrillar structure (Figure 9c). Due to the presence of thin crystalline fibrils connected to the crystalline lamellae, the heat-elongated PP exhibits high stress after yielding, as shown in Figure 4b, where the decrease in stress after the yield point was slight at 110 °C.

## 4. Conclusions

Highly strong PP can be obtained via uniaxial heat elongation at an elongation ratio of 600%, with our elongated sample presenting a high yield stress of 266 MPa at room temperature, which was 9.5 times larger than that of the unelongated PP. The elastic modulus *E*′ and the αc-relaxation temperature were also much higher for the heat-elongated than the unelongated PP due to the suppression of crystalline chain motion. The *E*′ remained high up to 140 °C in the heat-elongated PP, while it decreased to nearly zero at around 100 °C in the unelongated specimen. SAXS and DSC measurements revealed that the heat-elongated PP had macroscopically arranged stacked crystalline lamellae connected by thin fibrils, and the lamellae were fragmented during stretching at small strains below the yield point. Due to crystalline chain motion suppression and lamellar fragmentation during stretching at small strains, the heat-elongated PP was found to exhibit high tensile properties even at high temperatures, achieving a yield stress at 120 °C of 60 MPa, which was 7.5 times larger than that of the unelongated PP. These high tensile properties at high temperatures are promising for applications in industry, automobile parts and medical devices, where high temperature resistance is required [67]. This highly strong heat-elongated PP is promising for recycling friendly PP without the need for external fillers or reinforcement. To the best of our knowledge, this strengthening mechanism involving lamellar fragmentation at small strains and crystalline chain motion suppression is a new concept derived from the temperature dependence of tensile properties and deformation behavior, revealed using thermal analysis with DMA and DSC, as well as SAXS measurements. This strengthening mechanism differs from that of highly strong heat-elongated HDPE and TPU, in which strengthening is attributed to the prevention of lamellar fragmentation during stretching [50,51]. Despite the lamellar fragmentation in PP, fracture of the specimen via the breaking of the stacked lamellar structure is suppressed due to the presence of thin crystalline fibrils and taut amorphous tie chains, which increases the break strain, resulting in higher strength than that of HDPE and TPU. This concept will be confirmed by analyzing the tensile and creep behaviors of the highly strong heat-elongated PP after thermal deterioration, which will be presented in a separate paper.

## Figures and Tables

**Table 1:** Structural parameters of the unelongated and heat-elongated PP.

	Δ*H* (J/g)	*T*m (°C)	Lamellar Thickness (nm)	Amorphous Thickness (nm)	Long Period (nm)
unelongated PP	108.5	162.2	9.1	5.2	14.3
heat-elongated PP	150.9	168.0	10.3	7.1	17.4

## Data Availability

The original contributions presented in this study are included in the article and Supplementary Materials. Further inquiries can be directed to the corresponding author.

## Supplementary Materials

The following supporting information can be downloaded at https://www.mdpi.com/article/10.3390/polym17243238/s1. Figure S1. Photograph of a heat stretching apparatus.
