# Supplementary material for: Temperature Dependence of Tensile Properties and Deformation Behavior in Highly Strong Heat-Elongated Polypropylene
Source: Polymers (Basel). 2025 Dec 5;17(24):3238. doi: 10.3390/polym17243238 (PMC12736527; doi:10.3390/polym17243238)
Supplement: Supplementary file 1 [file polymers-17-03238-s001.zip › polymers-3998428-supplementary.pdf]

Supplementary Materials

Temperature dependence of tensile properties and deformation behavior in highly strong heat-elongated polypropylene

Karin Onaka, Hiromu Saito

Department of Applied Chemistry, Tokyo University of Agriculture and Technology, Koganei-shi, Tokyo 184-8588, Japan

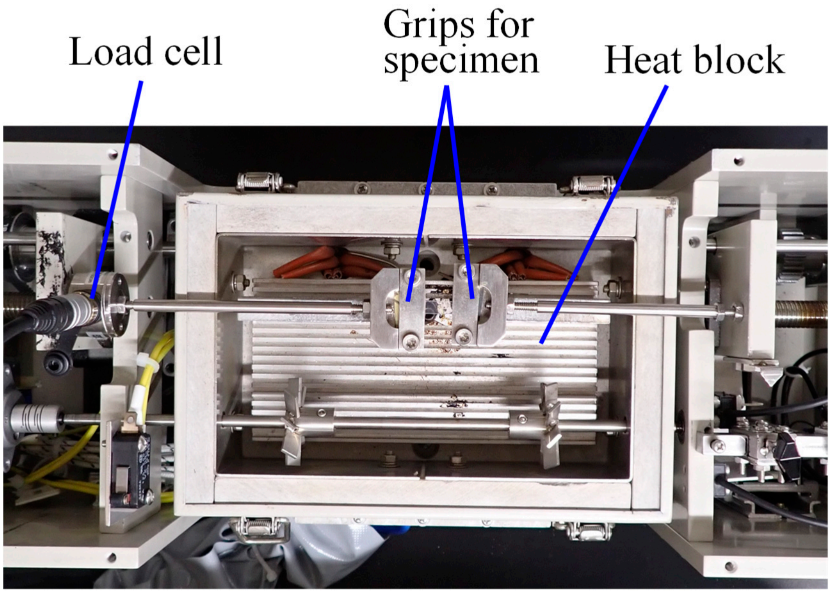

Figure S1. Photograph of a heat stretching apparatus.
